# Postoperative shoulder balance in Lenke type 1 adolescent idiopathic scoliosis patients with large thoracic curve (Cobb angle ≥ 70 degrees): a radiographic study

**DOI:** 10.1186/s12891-022-05554-9

**Published:** 2022-06-27

**Authors:** Jun Jiang, Xu Chen, Yong Qiu, Bin Wang, Yang Yu, Ze-zhang Zhu

**Affiliations:** Department of Orthopedic Surgery, Division of Spine Surgery, Nanjing Drum Tower Hospital, The Affiliated Hospital of Nanjing University Medical School, Zhongshan Road 321, Nanjing, 210008 China

**Keywords:** Idiopathic scoliosis, Thoracic, Radiographic, Shoulder

## Abstract

**Background:**

Large amounts of thoracic curve correction had been considered as a risk factor for postoperative shoulder imbalance (PSI) in adolescent idiopathic scoliosis (AIS) patients. This study aims to evaluate postoperative shoulder balance in Lenke type 1 AIS patients with large thoracic curve (Cobb angle ≥ 70 degrees) and compared it with those with moderate thoracic curve (Cobb angle < 70 degrees).

**Methods:**

A total of 47 Lenke type 1 AIS patients underwent posterior correction surgery between Sept. 2016 to Nov. 2018 in our institution were included. All these patient were divided into 2 groups based on the severity of main thoracic (MT) curve. Group A consisted of 25 cases with MT curve equal to or more than 70 degree while Group B consisted of 22 cases with MT curve less than 70 degree. Proximal thoracic (PT) Cobb angle, MT Cobb angle, MT apical vertebral translation (AVT), T2-T5 kyphosis, T5-T12 kyphosis, and radiographic shoulder height (RSH) were compared between these 2 groups preoperatively, immediately after surgery, and at a minimum of two-year follow-up.

**Results:**

Although all the correction of PT Cobb angle (15.8° ± 6.0° vs 12.5° ± 3.6°, *P* = 0.028), that of MT Cobb angle 47.3° ± 9.1° vs 30.9° ± 6.7°, *P* < 0.001) and that of MT AVT (35.1 mm ± 16.0 mm vs 24.1 mm ± 8.9 mm, *P* = 0.007) were significantly larger in Group A when compared with Group B, RSH was comparable between these 2 groups at last follow up (7.5 mm ± 7.4 mm vs 9.2 mm ± 4.2 mm *P* = 0.363). Most of the patients gained satisfactory shoulder balance with only 7 cases with minimal PSI in group A (28%) and only 6 cases with minimal PSI in group B (27.3%) at last follow-up (*P* > 0.05).

**Conclusions:**

Although Lenke type 1 AIS patients with large thoracic curve had more amounts of MT curve correction when compared with those with moderate thoracic curve, it did not lead to higher incidence of PSI if the correction rate is proper.

## Background

Achieving a symmetric shoulder is key to the evaluation of the surgical success in adolescent idiopathic scoliosis (AIS) patients because postoperative shoulder imbalance (PSI) has negative influence on patients’ cosmetic appearance and self-esteem [1–3]. Several risk factors have been postulated to be related to PSI in AIS patients, including a structural proximal thoracic (PT) curve, improper selection of upper instrumented vertebra (UIV), over-correction of the main thoracic (MT) curve, and so on [4–6].

Prior works had emphasized the importance of proper selection of UIV in preventing PSI [7, 8]. In theory, correction of right MT curve will elevate the left shoulder while correction of PT curve will bring down the left shoulder. Therefore, most of these authors suggested a proximal UIV (T3 or above) in patients with leveled or left higher shoulder because choosing a more cranial UIV allowed the spine surgeons better control the PT curve correction and can thus press the left shoulder down [9, 10]. For patients with Lenke type 1 curve, Trobisch suggested T2 as UIV if left shoulder is high, T3 if shoulders are level, and T4 if right shoulder is high [9]. Rose also reported guidelines to include T2 as UIV in patients with a left-elevated shoulder, T4 or even T3 in patients with leveled shoulder and T4 or T5 in patients with a right-elevated shoulder [10]. However, most recently, several studies had reported that the selection of UIV did not affect postoperative shoulder height in AIS patients. Hiett reported that the amount of MT curve correction was the only significant factor associated with postoperative shoulder balance in AIS patients [11]. Moorthy insisted that both greater percentage of MT curve correction and lower postoperative MT curve were independent risk factors for PSI while the UIV selection was not [12]. Sielatycki found that simply fusing a more proximal level did not reduce the odds of PSI in Lenke type 1 or 2 AIS patients. [13].

Nowadays, with the application of more powerful instrumentation, such as pedicle screw in the surgical treatment of AIS patients, obtaining a straight spine in AIS patients with a large MT curve became possible. However, in theory, abundant correction of MT curve might place these patients at a high risk of experiencing residual left-elevated shoulder after surgery. In addition, a larger thoracic curve may imply a more proximal UIV in AIS patients. Until now, the comparison of postoperative shoulder height between AIS patients with different severity of MT curve had not been investigated in previous studies. The purpose of the current study is to clarify whether AIS patients with a severe MT curve are more likely to have PSI than those with moderate MT curve after correction surgery.

## Methods

### Subjects

With approval from the institutional review board in our hospital, the AIS patients underwent posterior correction surgery from Sept. 2016 to Nov. 2018 in our institution were retrospectively reviewed. The inclusion criteria were: 1) with Lenke type 1 curve (single right main thoracic curve with proximal thoracic curve < 25° on side-bending X-ray films); 2) without previous treatment before surgery; 3) with a minimum follow-up of 2 years. Finally, a total of 47 cases met the criteria mentioned above were included. All these cases were further divided into 2 groups according to the magnitude of MT curve. There were 25 cases (21 females and 4 males) with MT curve equal to or more than 70 degrees (range,70 degrees to 115 degrees) in Group A with an average of 16.8 years old while there were 22 cases (19 females and 3 males) with MT curve less than 70 degrees (range,42 degrees to 62 degrees) in Group B with an average of 15.0 years old. There were 24 cases with lumbar modifier of A and 1 case with lumbar modifier of B in Group A while there were 21 cases with lumbar modifier of A and 1 case with lumbar modifier of B in Group B. In Group A, the UIV was located at T1 in 2 cases, T2 in 6 cases, T3 in 9 cases and T4 in 8 cases. In Group B, the UIV was located at T2 in 1 case, T3 in 4 cases, T4 in 13 cases and T5 in 4 cases. All these subjects were followed up for a mean of 2.4 years (2 years to 6 years). Informed consent was obtained for each participating subject.

### Surgical techniques

In Group B, we followed Lenke’s recommendations for selecting UIV [10]. The UIV was selected at T4 or below in patients with preoperative right-elevated shoulder, T3 in patients with leveled shoulder and T2 in patients with left-elevated shoulder. In group A, no one had upper end vertebra (UEV) located below T4 and most of them had UEV located at at T3 or above because the MT curve is very large. Hence, patients in Group A were proximally fused to UEV although they all had preoperative right-elevated shoulder.

The lower instrumented vertebra (LIV) was chosen at last substantial touching vertebra (LSTV) [14]. Under general anesthesia, the patient was placed in a prone position on the operation table. After a standard midline incision, the posterior parts of the spine were exposed with sub-periosteal dissection laterally to the transverse process. Pedicle screws were placed with a free hand technique. If the pedicle of thoracic vertebra was very small, the extrapedicular technique of screw placement was used. The lateral wall of the thin pedicle was intentionally breached and the screw passed through the pedicle-rib junction with the tip of the screw within the vertebral body. All the pedicle screws were polyaxial long-tab reduction screws, which facilitate capturing of the rod within the screw heads. After pedicle screw placements, the assistant push the scoliotic spine on the convex side and a pre-shaped rod with normal thoracic kyphosis was attached to the upper and lower screws on the concave side. The lower screw was firstly fully tightened, then the rest of the anchor points were gradually captured from the bottom to the top, which provided axial translation of the spine. Then the convex rod was also inserted. After finishing the rods insertion on both two sides, additional distraction was applied on the concavity while additional compression was applied on the convexity to realize the horizontalization at each level. Finally, left hand was putted on the left acromioclavicular joint and the right hand was putted on the right acromioclavicular joint to evaluated the shoulder balance. If the left hand is higher than the right hand (residual left-elevated shoulder), compression force was applied on the mostly proximal 2 to 3 screws on the left side to bring down the left shoulder. The neurophysiological monitoring were continuously performed during the operation.

### Radiographic measurements

The standing posteroanterior and sagittal X-ray films of the whole spine taken before surgery, immediately after surgery and at the last follow up were obtained for measurements in all cases. All these patients had the fists on on ipsilateral clavicles with elbows fully flexed when taking the sagittal X-ray films examination. The parameters assessed included the following [15]:1) proximal thoracic (PT) Cobb angle, MT Cobb angle, MT apical vertebral translation (AVT), T2-T5 sagittal Cobb angle, T5-T12 sagittal Cobb angle, and radiographic shoulder height (RSH): the difference in the soft tissue shadow directly superior to the acromioclavicular joint [6]. The RSH was defined as positive when the left shoulder was higher and negative when the right shoulder was higher. PSI was defined as RSH more than 10 mm and was further graded as significant imbalance (> 3 cm), moderate imbalance (2–3 cm), and minimal imbalance (1–2 cm) [6]. All these parameters were measured twice and averaged by the first author (JJ) using the software of Surgimap version 2.0 (New York, USA).

### Statistical analysis

Statistical analysis was performed by SPSS 14.0 software (Chicago, IL, USA). The Shapiro–Wilk test was used to test the data for a normal distribution. The parameters measured before surgery, immediately after surgery and at the last follow up were compared between these 2 groups by the independent-t test. The incidence of PSI between these 2 groups was compared by chi-square test. Correlation analysis was used to determine a Pearson coefficient (r) between preoperative parameters and RSH at last follow-up, as well as the change of RSH and the changes of the other parameters at last follow-up in all cases. Significance was established at the *P* < 0.05 level. After the initial correlation analysis, factors with a value of *P* < 0.05 were entered into a stepwise multiple regression analysis and the coefficient of multiple determination ( R^2^) was calculated.

## Results

All these radiographic parameters were normally distributed in both 2 groups. No significant difference was found between these 2 groups with respect to either mean age (*p* = 0.102) or sex distribution (*p* = 0.820). Patients in Group A had larger average PT Cobb angle (*P* < 0.001), average MT Cobb angle (*P* < 0.001), average MT AVT (*P* < 0.001) and average T5-T12 sagittal Cobb angle (*P* < 0.007) but smaller average RSH (*P* = 0.01) than those in Group B before operation (Table 1). All of these patients in Group A had right higher shoulder while 17 cases (77.3%) in Group B had right higher shoulder.

**Table 1:** The comparison of preoperative parameters between 2 groups

	**Group A (*n* = 25)**	**Group B (*n* = 22)**	***P* value**
Age (yrs)	16.8 ± 4.7	15.0 ± 2.6	0.102
Sex (F/M)	21/4	19/3	0.820
PT Cobb angle (°)	39.5 ± 6.9	30.4 ± 5.1	< 0.001*
MT Cobb angle(°)	76.6 ± 9.9	49.9 ± 7.1	< 0.001*
MT AVT (mm)	61.8 ± 18.7	38.9 ± 10.3	< 0.001*
T2-T5 kyphosis(°)	14.8 ± 6.5	14.4 ± 4.8	0.815
T5-T12 kyphosis(°)	27.2 ± 16.0	16.0 ± 10.1	0.007*
RSH (mm)	-16.3 ± 11.3	-7.7 ± 10.6	0.010*

*PT* proximal thoracic, *MT* main thoracic, *AVT* apical vertebral translation, *RSH* radiographic shoulder height, *means the difference was statistically significant

Although patients in Group A had comparable PT curve correction rate (*p* = 0.897), MT curve correction rate (*p* = 0.776) and MT AVT correction rate (*p* = 0.092) than those in group B, all the amount of PT curve correction (*p* = 0.006), amount of MT curve correction (*p* < 0.001) and amount of MT AVT correction (*p* = 0.005) were significantly larger in Group A when compared with Group B immediately after surgery (Table 2). The RSH was comparable between these 2 groups immediately after surgery (*p* = 0.839, Table 2).

**Table 2:** The comparison of correction outcomes between 2 groups

	**Group A (*n* = 25)**	**Group B (*n* = 22)**	***P* value**
**Immediately after surgery**			
PT Cobb angle (°)	21.6 ± 6.2	16.7 ± 5.1	< 0.001*
PT correction (°)	17.8 ± 5.9	13.6 ± 3.7	0.006*
PT correction rate(%)	44.9 ± 13.6	45.4 ± 12.0	0.897
MT Cobb angle(°)	26.5 ± 10.4	17.5 ± 5.2	0.001*
MT correction (°)	50.0 ± 9.5	32.4 ± 6.8	< 0.001*
MT correction rate(%)	65.7 ± 11.7	64.8 ± 11.2	0.776
MT AVT (mm)	24.8 ± 14.0	11.9 ± 7.3	< 0.001*
MT AVT correction (mm)	37.0 ± 13.1	27.0 ± 9.6	0.005*
MTAVT correction rate (mm)	60.4 ± 16.8	69.1 ± 18.0	0.092
T2-T5 kyphosis(°)	16.6 ± 5.1	14.4 ± 4.3	0.108
T5-T12 kyphosis(°)	22.8 ± 6.8	21.0 ± 6.4	0.368
RSH (mm)	6.0 ± 6.4	6.4 ± 5.8	0.839
RSH change (mm)	22.3 ± 13.7	14.0 ± 10.0	0.025*
**At last follow-up**			
PT Cobb angle (°)	23.7 ± 6.2	17.9 ± 5.1	0.001*
PT correction (°)	15.8 ± 6.0	12.5 ± 3.6	0.028*
PT correction rate(%)	39.5 ± 13.8	41.5 ± 12.8	0.623
MT Cobb angle(°)	29.3 ± 10.3	19.0 ± 4.9	< 0.001*
MT correction (°)	47.3 ± 9.1	30.9 ± 6.7	< 0.001*
MT correction rate(%)	62.1 ± 11.3	61.8 ± 10.0	0.917
MT AVT (mm)	26.7 ± 12.4	14.7 ± 6.5	< 0.001*
MT AVT correction (mm)	35.1 ± 16.0	24.1 ± 8.9	0.007*
MTAVT correction rate (mm)	56.5 ± 17.7	24.1 ± 8.9	0.295
T2-T5 kyphosis(°)	17.0 ± 4.8	17.5 ± 4.8	0.724
T5-T12 kyphosis(°)	23.4 ± 5.1	22.5 ± 5.7	0.571
RSH (mm)	7.5 ± 7.4	9.2 ± 4.2	0.363
RSH change (mm)	23.8 ± 13.9	16.0 ± 10.2	0.060
Incidence of PSI	28.0% (7/25)	27.3%(6/22)	0.956

*PT* proximal thoracic, *MT* main thoracic, *AVT* apical vertebral translation, *RSH* radiographic shoulder height, *PSI* postoperative shoulder imbalance. *means the difference was statistically significant

At last follow up, all the correction rate of PT curve (*p* = 0.623), correction rate of MT curve (*p* = 0.917) and correction rate of MT AVT (*p* = 0.295) were also comparable between these 2 groups (Table 2). However, all the amount of PT curve correction (*p* = 0.028), amount of MT curve correction (*p* < 0.001) and amount of MT AVT correction (*p* = 0.007) were significantly larger in Group A when compared with Group B at last follow up (Table 2). No significant difference of RSH was found between these 2 groups at last follow up (*p* = 0.363,Table 2).

**Fig. 1:**
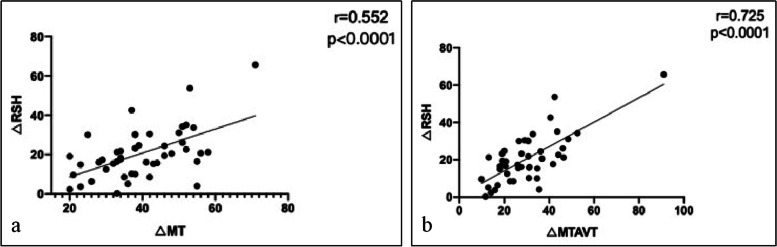
**a**-**b** Significant association between the MT correction and the change of RSH at last follow up (**a**). Significant association between the MT AVT correction and the change of RSH at last follow up (**b**)

The incidence of PSI was 28% (7/25) in Group A and was 27.3% (6/22) in Group B at last follow-up (*P* = 0.956, Table 2). All these 13 case with PSI had minimal shoulder imbalance. Correlation analysis demonstrated that none of these preoperative parameters was significantly associated with RSH at last follow up (Table 3). Both the correction of MT Cobb angle (*p* < 0.001) and that of MT AVT (*p* < 0.001) were significantly positively associated with that of RSH at the last follow-up in all these patients (Table 4, Fig. 1). Both 2 variables were then entered into stepwise multiple regression analysis, which revealed that only the correction of MT AVT was significant independent predictor of the change of RSH. The coefficient of multiple determination ( R^2^) of the MT AVT correction was 0.556, indicating that MT AVT correction explained 55.6% of the change of RSH at last follow up (Table 4).

**Table 3:** The correlations between the preoperative parameters and RSH at last follow up in all patients (*n* = 47)

Measurements	Correlation Coefficient ( r)	*P* value
PT curve (°)	-0.05	0.738
MT curve (°)	-0.067	0.654
MT AVT (mm)	0.035	0.814
T2-T5 kyphosis (°)	0.063	0.673
T5-T12 kyphosis (°)	-0.231	0.119
RSH (mm)	0.088	0.556

*PT* proximal thoracic, *MT* main thoracic, *AVT* apical vertebral translation, *RSH* radiographic shoulder height

**Table 4:** The correlations between the change of RSH and curve correction at last follow up in all patients (*n* = 47)

Measurements	Correlation Coefficient ( r)	Coefficient of Multiple Determination ( R^2^)
PT correction (°)	-0.083	
MT correction (°)	0.552^a^	
MT AVT correction (mm)	0.725^a^	0.556^a^
T2-T5 kyphosis correction (°)	-0.258	
T5-T12 kyphosis correction (°)	-0.244	

*PT* proximal thoracic, *MT* main thoracic, *AVT* apical vertebral translation, *RSH* radiographic shoulder height, ^a^means the difference was statistically significant

## Discussion

**Fig. 2:**
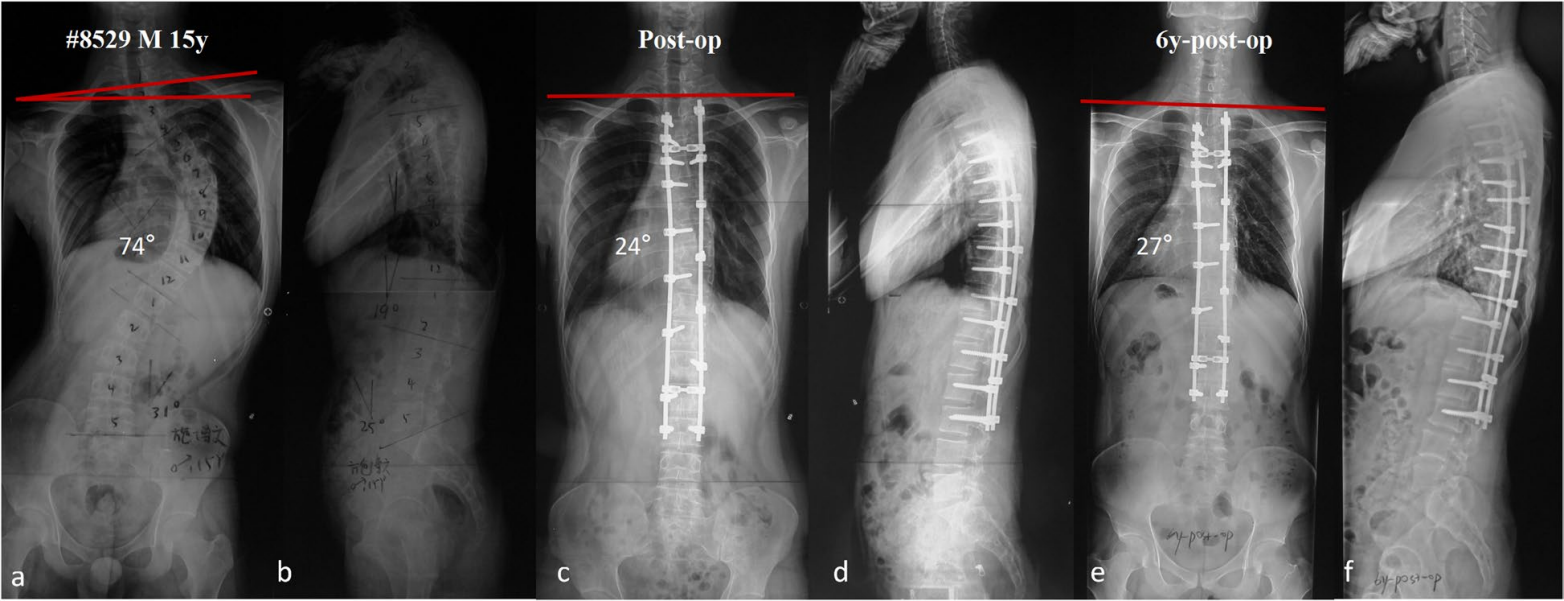
**a**-**f** A 15-year-old male patient with MT Cobb angle of 74°and preoperative RSH of -24.7 mm (**a-b**). This patient was proximally fused to T3 with MT Cobb angle corrected to 24°and the RSH improved to 7.7 mm immediately after surgery (**c**-**d**). The MT Cobb angle was 27°and the RSH was 9 mm 6 years after surgery in this patient with shoulder balance well maintained(**e**–**f**)

**Fig. 3:**
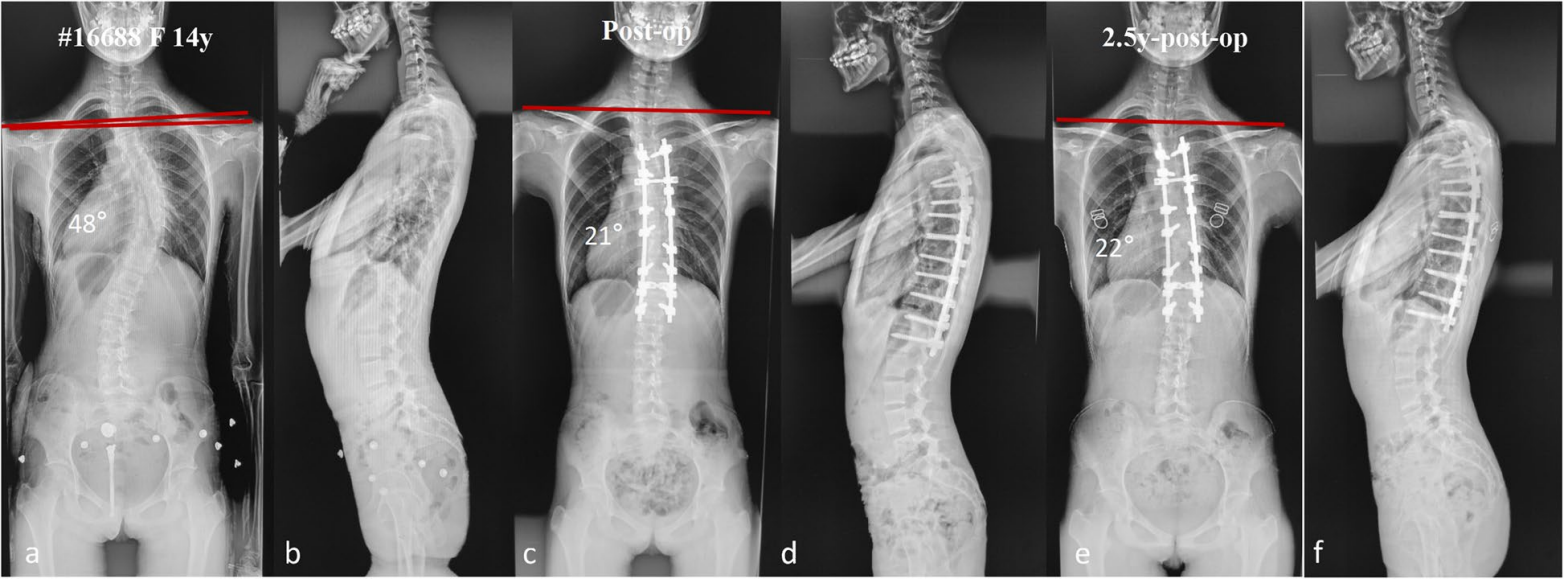
**a**-**f** A 14-year-old female patient with MT Cobb angle of 48°and preoperative RSH of -4.3 mm (**a**-**b**). This patient was proximally fused to T4 with MT Cobb angle corrected to 21°and the RSH changed to 1 mm immediately after surgery (**c**-**d**). The MT Cobb angle was 22°and the RSH was 2 mm at the last follow-up in this patients with satisfactory shoulder balance (**e**–**f**)

In the current study, we firstly compared the postoperative shoulder balance between AIS patients with different severity of MT curve. Both inadequate UIV and over-correction of MT curve had been considered to be associated with PSI in AIS patients. However, in our patients, although patients in Group B had lower UIV, the postoperative RSH is similar between these 2 groups. The UIV seems not to be an independent factor for postoperative shoulder height in AIS patients. Additionally, since the over-correction of MT curve can drive the contralateral shoulder to imbalance, theoretically a lager preoperative MT curve may correspond with an increased likelihood for PSI in AIS patients, especially when more powerful instrumentation, such as pedicle screws are used. The results of our study demonstrated that although patients with severe curve had larger MT curve corrections, they had comparable postoperative RSH when compared with those with moderate curve (Figs. 2 and 3). Most of these patients had satisfactory postoperative shoulder balance and no patients had moderate or significant PSI in both 2 groups.

In our opinion, postoperative shoulder balance is determined by both preoperative shoulder height and intraoperative correction maneuver. One of our previous studies found that preoperative directionality of shoulder mainly depends on the profile of MT curve in Lenke type 2 AIS patients [16]. Since a right MT curve can elevate the right shoulder, patients with larger MT curve had more right higher shoulder than those with smaller MT curve. In this study, we found that such phenomenon also existed in Lenke type 1 patients. Patients in Group A had significantly larger average MT Cobb angle but smaller average RSH (more right higher shoulder) than those in Group B before operation (*P* < 0.05). Although the MT correction rate was similar between these 2 groups, the amounts of MT correction was significantly higher in patients in Group A since they had larger preoperative MT curve. The large amounts of MT curve led to more left shoulder elevation. However, this would not led to higher incidence of PSI in patients with large curve since they had more preoperative right-elevated shoulder. The correlation analysis of our study also demonstrated that no preoperative parameter can predict the postoperative RSH.Therefore, we conclude that a more severe MT curve with a right higher shoulder does not necessarily imply an increased risk of PSI in Lenke type 1 AIS patients.

**Fig. 4:**
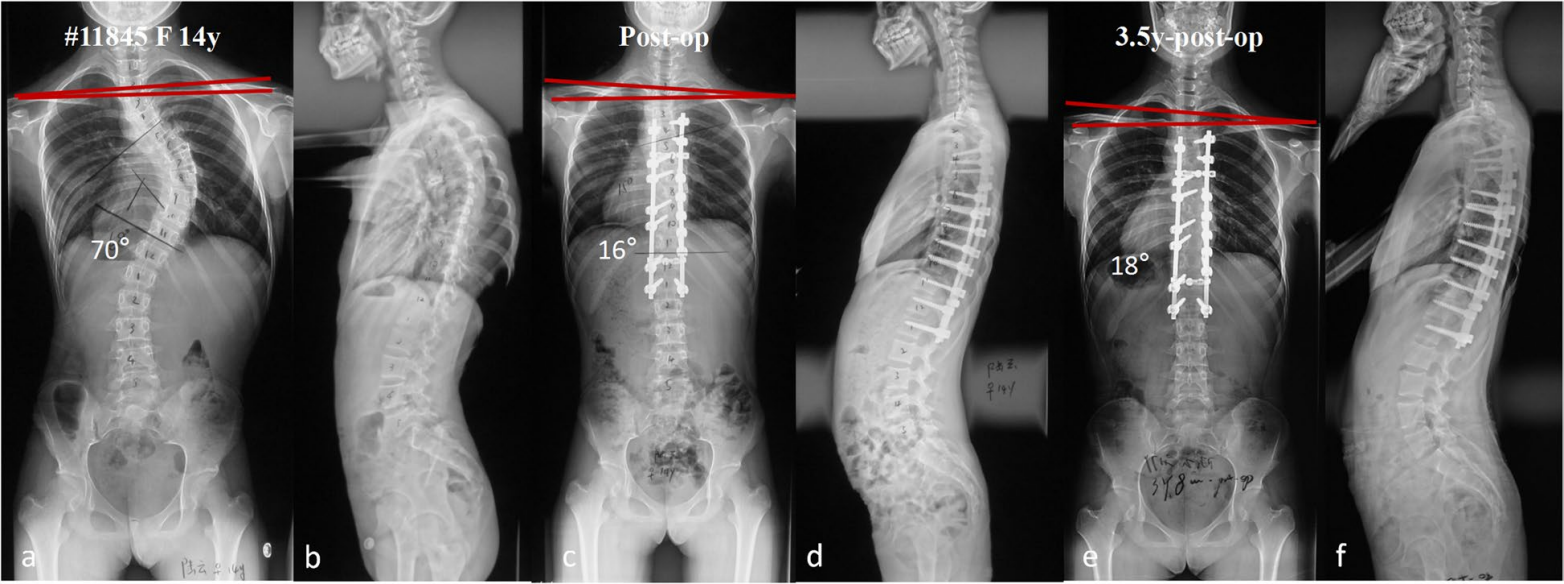
**a**-**f** A 14-year-old female patient with MT Cobb angle of 70°and preoperative RSH of -10.1 mm (**a**-**b**). This patient was proximally fused to T4 with MT Cobb angle corrected to 16°and the RSH changed to 13.1 mm immediately after surgery (**c**-**d**). The MT Cobb angle was 18°and the RSH was 12.5 mm at the last follow-up (**e**–**f**). This patient had minimal PSI due to the overcorrection of MT curve with correction rate of 77.1% immediately after surgery and 74.3% at the last follow up

**Fig. 5:**
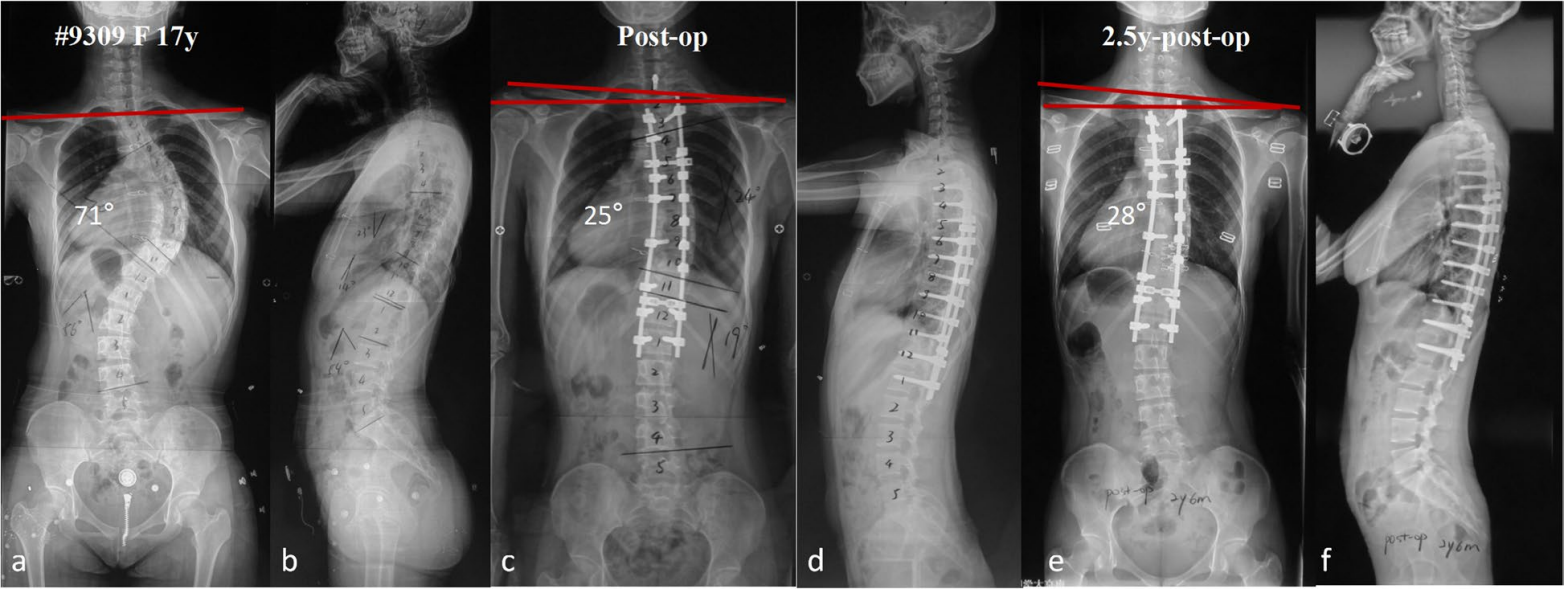
**a**-**f** A 17-year-old female patient with MT Cobb angle of 71°and preoperative RSH of -2.2 mm (**a**-**b**). This patient was proximally fused to T3 with MT Cobb angle corrected to 25°and the RSH changed to 11.9 mm immediately after surgery (**c**-**d**). The MT Cobb angle was 28°and the RSH was 15.3 mm at the last follow-up in this patients (**e**–**f**). This patient still had PSI without overcorrection of MT curve due to preoperative mild right elevated shoulder with insufficient selection of UIV (T3 indicates PT curve partially fused)

Although large MT curve might not lead to residual shoulder imbalance in our patients, the spine surgeons cannot ignore the necessity of avoiding excessive curve correction during the operation. In fact, the average correction rates of MT curve were only 65.7% immediately after surgery and 62.1% at last follow-up in Group A. Over-correction of MT curve is still a risk factor for PSI in these patients. The correlation analysis demonstrated that both MT correction and MT AVT correction had positively significant associations with the change of RSH at last follow up. The multiple regression analysis showed that MT AVT correction was a significant independent predictor of the change of RSH. For example, one patient in Group A still had PSI with the MT correction rate up to 77.1% (Fig. 4). Furthermore, we also should be aware of that preoperative shoulder height does not solely depends on the severity of MT curve [16]. Not all the patients with severe MT curve had marked right higher shoulder. In our study, there were 6 cases (24%) with absolute value of preoperative RSH less than 10 mm in Group A. For these patients with large MT Cobb angle and mild right higher shoulder, sole MT correction (proximally fused to T3 or below) might lead to a residual left-elevated shoulder after surgery (Fig. 5). Full fusion of PT curve (proximally fused to T2 or above) is suggested in these patients so that surgeons can correct PT curve as much as possible to compensate the effect of left shoulder elevation from MT correction.

There were several limitations should be mentioned in the current study. Firstly, the sample size is relatively small. Secondly, a few studies had noted the discrepancy between medial shoulder and lateral shoulder. Parameters reflecting medial shoulder height were not investigated in our study. Thirdly, it is difficult to ensure all the patients had the full-length spine X-ray film examination in the same standard position, which might influence the radiographic measurements.

## Conclusion

This is the first study comparing postoperative shoulder balance between Lenke type 1 patients with different severity of MT curve. The results indicated that a large preoperative MT Cobb angle did not necessarily imply a high incidence of PSI in these patients. However, when treating patient with large MT curve, surgeons still need to take other risk factors for PSI into account, such as excessive MT curve correction, insufficient selection of UIV, and so on, to make the most reasonable surgical plan for these patients.

## Data Availability

The data and materials in current paper may be made available upon request through sending an e-mail to first author.

## Abbreviations

PSI: Postoperative shoulder imbalance
AIS: Adolescent idiopathic scoliosis
MT: Main thoracic
PT: Proximal thoracic
AVT: Apical vertebral translation
RSH: Radiographic shoulder height
UIV: Upper instrumented vertebra
UEV: Upper end vertebra
LIV: Lower instrumented vertebra
LSTV: Last substantial touching vertebra
